# Mobile teledermatology for skin cancer screening: A diagnostic accuracy study

**DOI:** 10.1097/MD.0000000000006278

**Published:** 2017-03-10

**Authors:** Stefan Markun, Nathalie Scherz, Thomas Rosemann, Ryan Tandjung, Ralph P. Braun

**Affiliations:** a Institute of primary care, University and University Hospital of Zürich, Zürich, Switzerland; b Department of Dermatology, University and University Hospital of Zürich, Zürich, Switzerland.

**Keywords:** m-health, screening, sensitivity, skin cancer, specificity, teledermatology

## Abstract

Skin cancer screening has undoubted potential to reduce cancer-specific morbidity and mortality. Total-body exams remain the prevailing concept of skin cancer screening even if effectiveness and value of this method are controversial. Meanwhile, store and forward teledermatology was shown to be a reliable instrument for several diagnostic purposes mostly in specialized dermatology settings. The objective of this study was to evaluate most convenient mobile teledermatology interventions as instruments for skin cancer screening in a representative population.

Prospective diagnostic study with visitors of a skin cancer screening campaign in Switzerland. Histopathology was used as reference standard. Mobile teledermatology with or without dermoscopic images was assessed for performance as a screening test (i.e., rule-in or rule-out the need for further testing). Outcomes were sensitivity, specificity, and predictive values.

Seven cases of skin cancer were present among 195 skin lesions. All skin cancers were ruled-in by teledermatology with or without dermoscopic images (sensitivity and negative predictive value 100%). The addition of dermoscopic images to conventional images resulted in higher specificity (85% vs. 77%), allowing reduction of unnecessary further testing in a larger proportion of skin lesions.

Store and forward mobile teledermatology could serve as an instrument for population-based skin cancer screening because of favorable test performance.

## Abstract 2

Supplemental Digital Content is available in the text

## 1 Introduction

Skin cancer is on the rise globally and especially so in Switzerland.^[1]^ Campaigns to raise awareness and foster prevention and early detection of skin cancer are ongoing since over a decade.^[2,3]^ Owing to superficial growth and natural course running through preclinical stages, skin cancer is highly amenable for screening interventions with potential economical and health-benefits.^[4,5]^ The selection of the population to screen, the kind of healthcare professionals to appoint, and the type of clinical assessment to perform, however, are still to debate. Moreover, the effectiveness of skin cancer screening in general still remains to be determined.^[6]^ So far, the prevailing concept of skin cancer screening is the total-body exam executed by trained physicians. This type of intervention, however, is very expensive and possibly disproportional to introduce on a population-based scale. The nationwide skin cancer screening program in Germany, for instance, relied on total-body exams but surprisingly failed to result in improvements of skin cancer mortality rates despite promising results from a pilot project.^[7,8]^ The role of total-body exams might be the one of a case-finding procedure in an at-risk population, rather than a population-based screening intervention. In contrast, lesion directed examinations are more time-efficient and have similar detection rates, but still require time-expensive face-to-face consultations and similar constraints as to total-body examinations apply.^[9]^ Teledermatological assessments on the other hand, especially lesion directed store and forward procedures can be performed in a most speedy manner. In several former studies, the reliability of teledermatology based management recommendations was shown to be comparable to face-to-face consultations.^[10–15]^ Moreover, recent technical advancements led to mobile phone assisted dermoscopy even enabling “mobile teledermoscopy,” combining high technical standards of teledermatological image acquisition with mobile phone convenience. Feasibility and reliability for cancer detection with such mobile teledermoscopy has already been shown in high prevalence settings.^[16,17]^

Given time-efficiency, reliability, and the widespread dissemination of powerful digital cameras integrated in mobile phones, the remaining technical limitations to mobile teledermatology for skin cancer screening are few and may lie in standardization of the specific methods of image acquisition to achieve highest possible reliability.^[18]^ Once these issues are resolved, image acquisition could be transferred to settings outside medical practices and broader population based screening could be implemented.

To assess a screening intervention in this context, test performance measures (sensitivity, specificity, and predictive values) in directing malignant lesions to further evaluation (and treatment) and withholding these measures in non-malignant skin lesions are needed to know. Also, the detection rate of malignant skin lesions (i.e., number needed to screen) in a representative low-prevalence setting is relevant to estimate the value of the screening intervention. So far, however, only little research in mobile teledermatology has been conducted in settings representative for population based screening, and research concentrated on specialized secondary or tertiary care settings. Furthermore, the additional value of teledermoscopy on top of conventional teledermatology for screening interventions is still unclear.

Therefore, the aim of this study was to estimate the diagnostic performance (sensitivity, specificity, and predictive values) of lesion-directed mobile teledermatology with or without dermoscopic imaging as a skin cancer screening intervention in a population representing a low-prevalence setting.

## 2 Methods

### 2.1 Study design

We conducted a prospective diagnostic study with data collection during a public skin cancer screening campaign at the Department of Dermatology at the University Hospital of Zürich in May 2013. Ethics committee of the Canton of Zürich approved the study on May 8, 2013 (KEK-ZH-Nr. 2013–0119). The study was conducted according to the tenants of the declaration of Helsinki and to good clinical practice standards.

### 2.2 Screening campaign and participants

The “National skin cancer day” is a yearly conducted skin cancer screening campaign in Switzerland and is carried out in dermatology clinics and hospitals since 2006. Anyone interested may visit a participating site without scheduled appointment to present specific skin lesions to be checked for malignancy (total-body exams are not performed). In order to reduce administrative expenditures during the campaign, participants do not register at the clinic but are guided through the screening anonymously without any information being documented. For the purpose of this study and to evaluate quality of the procedures during the campaign, we prospectively documented skin lesions with digital images and structured clinical information and followed outcomes of biopsies.

At the reception, participants were given consecutive numbers in order of their appearance in the clinic. Participants were then allocated to 1 of 18 examination rooms. In 8 of the 18 examination rooms, personnel and camera equipment for study participation was available and participants were approached for study participation. The allocation to the 18 examination rooms was performed consecutively in order of appearance at the reception and according to availability in next possible examination room. Thereby, we sought to prevent selection of specific skin lesions for specific examination rooms. We included skin lesions of participants that provided informed consent and the documentation of the skin lesion with macroscopic and microscopic digital images succeeded.

### 2.3 Clinical measures and skin cancers

The lesion-directed clinical examinations were performed by residents of the Department of Dermatology with an on-call backup by senior staff for assistance in uncertain cases. From examinations, the following data were filled in a standardized form: age, sex, personal or family history of skin cancer, skin type according to Fitzpatrick, the estimated total number of moles (>100 or <100), suspected diagnoses and management recommendations for the skin lesion under question.

When skin cancer was suspected, participants were offered appointments (immediately or within a few days) to remove the skin lesions or to perform biopsy. The biopsy results were available to us and served as the reference standard for skin cancer being present or not. When skin cancer was ruled out clinically, obviously no additional biopsy was performed and the clinical decision served as reference standard.

### 2.4 Teledermatology method and measures

#### 2.4.1 Image acquisition:

Medicine students who completed a 1-hour training workshop in skin lesion photography acquired 3 images of every skin lesion utilizing the integrated 5-megapixel camera of an iPod (Apple Inc.) touch fifth generation. First, an overview image that showed anatomical region of the lesion, second a macroscopic image from approximately 10 cm that showed the surrounding skin including the dispersion of other skin lesions, third a dermoscopic image was taken using the Handyscope (FotoFinder Systems GmbH, Bad Birnbach, Germany) (purpose-built attachable dermoscope that converts an iPhone or iPod touch into a digital dermoscope for mobile skin examinations, allowing polarized and non-polarized images of up to 20 times magnification; the product was not Food and Drug Administration labeled for the use under discussion).^[19]^ All acquired images were JPEGs with a resolution of 2592 × 1936 pixels.

#### 2.4.2 Teledermatological assessment:

The images were arranged by skin lesions and organized in 2 packages, the first package including macroscopic images only and the second package including the additional teledermoscopic images. The packages were stored on encrypted hard-drives and sequentially forwarded to the teledermatologist (see acknowledgment) with a 1-month washout time interval between the first and the second package. The teledermatologist was a board certified dermatologist and very experienced in the teledermatological examination of skin lesions including digital teledermoscopy. Along with the images, the teledermatologist was given the information about the patients’ age, sex, history of skin cancer, Fitzpatrick skin type, and the estimated number of moles retrieved from the questionnaire of the clinical examination. The teledermatologist was aware of her task being skin cancer screening but was left unaware of the aim of the study and blinded towards suspected diagnoses and management recommendations already given by the dermatologists at the screening campaign and towards biopsy results. For every skin lesion, the teledermatologist first decided whether the provided images were adequate to allow assessment for cancer screening at all. Secondly, in adequate cases, management recommendations had to be given in 3 categories: (a) no follow-up required, (b) clinical assessment required, (c) direct excision or biopsy.

### 2.5 Statistical analysis

Quantitative variables are reported as medians and interquartile ranges, categorical variables are reported as counts and proportions. Management recommendations from teledermatology were dichotomized as follows: “no follow-up required” was categorized as negative outcome of the screening test and “clinical assessment required” or “direct excision or biopsy” was categorized as positive test outcome. Skin lesions that had histopathology-confirmed skin cancers were categorized as cancer-positive, all others as cancer-negative.

We calculated the number of patients to be screened to detect a case of skin cancer, by dividing the total number of histology confirmed skin cancer by the number participants included in the study. Furthermore, we calculated test sensitivity, specificity, and predictive values in according to the test outcomes categorized above with reference to cancer being present or not according to the biopsy confirmed results. Agreement between teledermatology and gold standard are expressed by concordance rate as well as Cohen's Kappa (*κ*). All analyses were performed in duplicate for the 2 test methods with or without teledermoscopic images.

### 2.6 Outcomes

The primary outcomes of this study were the test performance measures of teledermatology with and without teledermoscopy. Further outcomes were the practicability of the techniques measured by skin lesions the teledermatologist rated to be suitable to perform cancer screening, moreover as an estimate of potential value of the screening intervention we calculated the number of skin lesions needed to be screened to detect 1 case of skin cancer.

## 3 Results

### 3.1 Population

**Figure 1:**
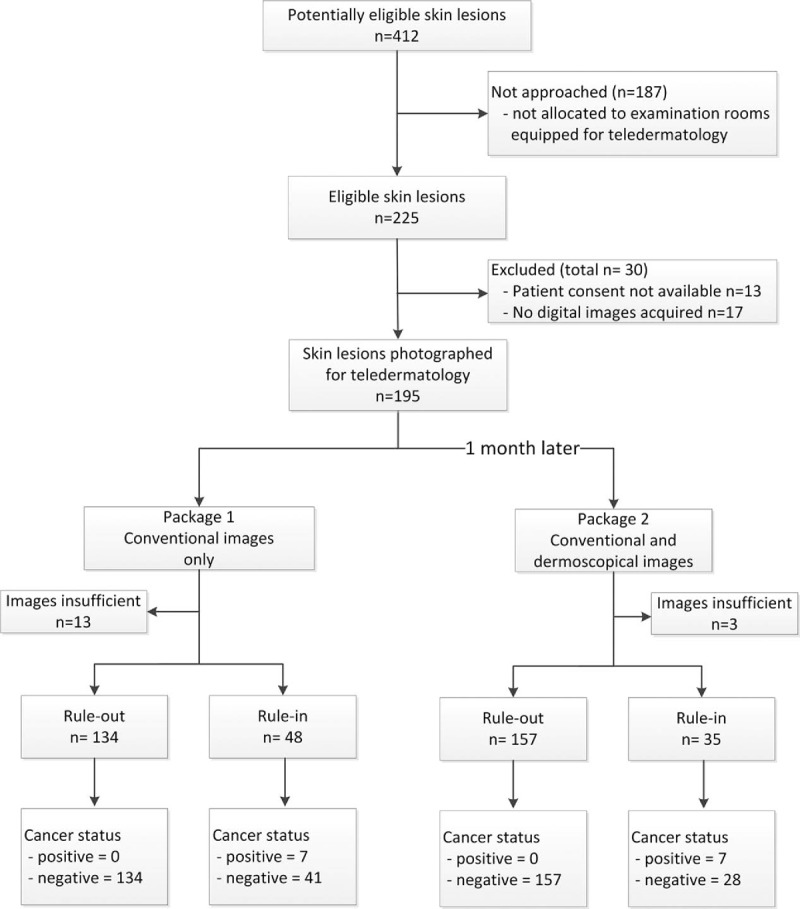
Study flow-chart and outcome counts.

A total of 412 skin lesions (in 405 patients) were counted at the screening campaign on May 13th, 2013 and 225 skin lesions were seen in examination rooms equipped for study participation. In 13 (5.8%) of these informed consent was unavailable and in 17 (7.6%) no digital images were acquired because of technical problems, thus resulting in 195 (86.7%) of the eligible skin lesions included in the study (Fig. 1).

Skin lesions in the study originated from a total of 188 patients, population characteristics are given in Table 1. The clinically suspected diagnoses and management recommendations for skin lesions are summarized in Table 2. The prevalence of malignancy in the sample was 3.6%.

**Table 1:**
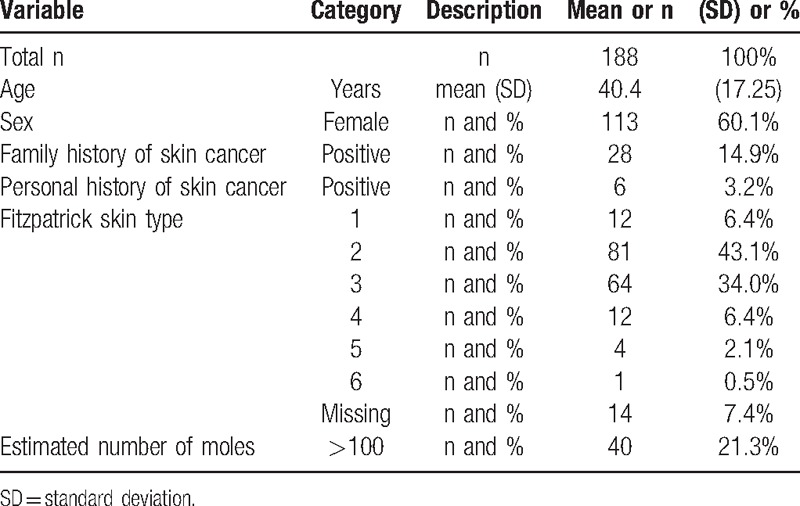
Characteristics of study participants at the screening campaign, total n = 188.

**Table 2:**
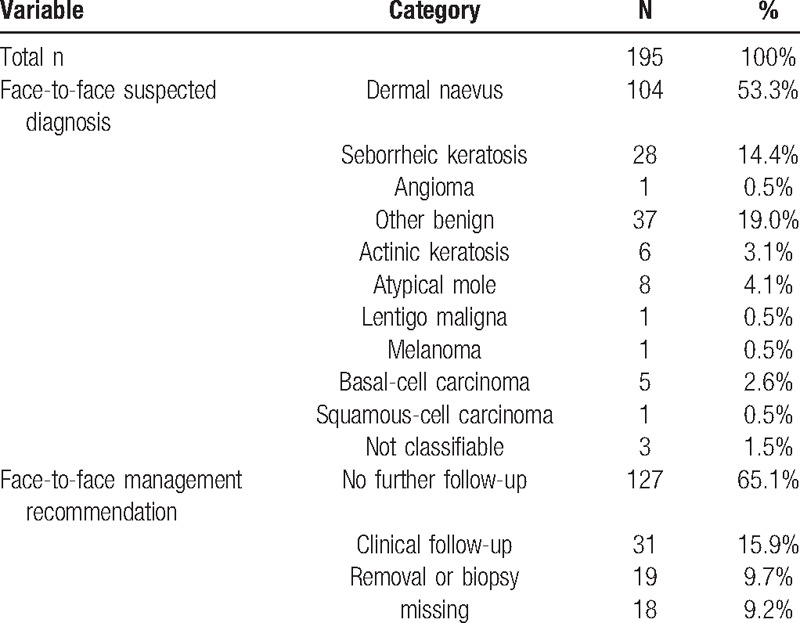
Characteristics of skin lesions at the screening campaign, total n = 195.

### 3.2 Practicability of teledermatology as screening test and number needed to screen

Conventional images alone were suitable to perform skin cancer screening in 182 (93.3%) of the cases, in the image package including teledermoscopic images 192 (98.5%) skin lesions were suitable. To detect 1 case of skin cancer, we needed to screen 27 skin lesions in our sample.

### 3.3 Performance of teledermatology as screening tests

Test performance measures of conventional teledermatology were 100% sensitivity, 76.6% specificity, 14.6% positive predictive value, and 100% negative predictive value. Conventional teledermatology was able to rule-out the need for any further follow-up in 134 of 182 skin lesions (73.6%) without missing any of the skin cancers in the sample. Overall concordance of conventional teledermatology with reference standard was 77.5% (*κ* = 0.20).

Additional teledermoscopy on top of conventional teledermatology performed with 100% sensitivity, 84.9% specificity, 20.0% positive predictive value, and 100% negative predictive value. The rule-out of any further follow-up was possible in 157 of 192 (81.8%) skin lesions without missing any of the skin cancers in the sample. Overall concordance of teledermatology featuring teledermoscopy with reference standard was 85.4% (*κ* = 0.29).

Detailed cross tabulation of test outcomes according to the reference standard is given (supplemental information files 1 and 2).

### 3.4 Additional exploratory analyses

In order to assess whether different levels of clinical presentations were associated with different test-performance, we applied a binomial logistic regression model with the dependent variable being the agreement of the teledermatology with reference standard. As independent variables, we added all clinical variables from the clinical assessment.

For teledermatology without teledermoscopic images we found no association of clinical presentations with test performance. In teledermatology including teledermoscopy, however, the clinical presentations of atypical mole and age were both associated with lesser odds for agreement with reference standard: atypical mole odds ratio for agreement (OR) = 0.05 (95% confidence interval [CI] = 0.001–0.28); age OR = 0.96 (95% CI = 0.92–0.99).

## 4 Discussion

In this study, mobile teledermatology detected all cases of skin cancer in a low prevalence population and accurately ruled out the necessity of further testing in 3 out of 4 cases. Mobile teledermoscopy in addition to conventional images increased specificity of the screening allowing correct rule-out in 4 out of 5 skin lesions. Mobile teledermoscopy also had higher practicability featuring a substantially lower rate of images classified as inadequate for screening compared with conventional mobile teledermatology.

Findings from our study are in line with previous research demonstrating very high cancer detection rates of mobile teledermoscopy in high-prevalence settings.^[16,17]^ In a very similar setting as ours, a high management concordance between face-to-face consultations and conventional teledermatology has recently been found and cancer screening utilizing teledermatology was suggested.^[20]^ Here, we followed the diagnostic chain beyond agreement with clinical management recommendations (suffering from interrater variability) and linked the results to a valid gold-standard represented by histopathology, therefore allowing test performance measurements in a screening population.

The specific methods in skin cancer screening are still to debate and our results emphasize teledermatology's potential contribution to this field: first, sensitivity and negative predictive value for ruling in skin cancers (to either subsequent clinical assessment or direct excision) was 100% clearly arguing for qualification of the technique as a screening intervention, because no case of skin cancer was falsely ruled out. Furthermore, only minimal training of photographers was needed to enable acquisition of images suitable for the teledermatologist arguing for practicability of the technique. We did not systematically assess the teledermatologist's time expenditures to perform the assessments; however, each set (consisting in almost 200 images) was finished within “one afternoon” strongly arguing for time-efficiency of the procedure considering that the image material was derived from face-to-face exams performed by 8 physicians working an entire day each. Looking at the additional value of teledermoscopy on top of conventional teledermatology, we found that more skin lesions were suitable to be screened but more importantly, specificity increased allowing more lesions to be ruled-out by the screening with the exception of atypical moles. Thus, considering that additional expenses in technical equipment and time to perform 1 additional image are small, mobile teledermoscopy might be a valuable enhancement in addition to conventional mobile teledermatology because it can effectively decrease unnecessary follow-up exams due to false-positive screening outcomes.

Our results show how teledermatology could contribute to skin cancer screening by image acquisition also outside medical practice by minimally instructed personnel (possibly even patients themselves) as piloted for other clinical questions in dermatology.^[21]^ Similar procedures are by now only proposed and integrated in remote and medically under-supported areas but such a service might also decrease skin cancer rates in other healthcare settings when offered to the broad population.^[22]^ Even if total-body exams presumably have higher detection rate in 1 specific individual, the population-wide detection rate may be higher using a teledermoscopy service. This because of lower barriers and easier access to teledermatology than to total-body exams and because of already very high acceptance among the population.^[23]^ Notably, such a screening would ultimately depend on self-detection of skin lesions by individuals among the population. The comparably high cancer rate we found in our population speaks in favor of the population's ability for self-detection, furthermore this ability has specifically been found before in a comparable screening setting.^[24]^

### 4.1 Strengths and limitations

The main strength and novelty of our study is the head-to-head comparison of teledermatological skin cancer screening interventions in a representative population with a valid gold-standard available. Limitations to this study are the following: first, only 1 teledermatologist was involved and since there is no standardized training or generally accepted method of image acquisition and image evaluation, reproducibility of our results is uncertain. The low-level of efforts needed to implement image acquisition combined with the positive results per se, however, are encouraging for further pursuit of the concept. Secondly, teledermatology may still have missed skin cancers because histopathology was not available in every 195 skin lesion assessed, however, these hypothetical skin cancers would have also been missed by the face-to-face examinations. Therefore, teledermatology was at least non-inferior to face-to-face examinations that are themselves the commonly accepted standard in skin cancer screening.

### 4.2 Recommendations for further research

For further research we suggest that teledermatology's test performance is investigated depending on reproducible standards of image acquisition and image evaluation using skin lesions originating from a population representative for skin cancer screening. Especially, there might still be room left to improve specificity of the screening without renouncing sensitivity that appears already to be very high. Further steps then include research in delivery and design of a population-based teledermatology screening intervention (i.e., with image acquisition outside medical practices) before a population wide reduction of skin cancer burden can be attempted.

We conclude that store and forward mobile teledermatology is inexpensive, practicable, and able to produce highly accurate management recommendations in the specific role of skin cancer screening. Population-based skin cancer prevention programs could consider implementing mobile teledermatology services as screening tests rather than directly offering total-body exams.

## Supplementary Material

Supplemental Digital Content
