# Supplementary material for: Mobile teledermatology for skin cancer screening: A diagnostic accuracy study
Source: Medicine (Baltimore). 2017 Mar 10;96(10):e6278. doi: 10.1097/MD.0000000000006278 (PMC5348191; doi:10.1097/MD.0000000000006278)
Supplement: Supplemental Digital Content [file medi-96-e6278-s001.doc]

Supplemental table 1: Cross tabulation of conventional teledermatology test performance

| Index test: conventional teledermatology; Reference test: face to face encounter and histopathology; total valid n=182 | | | | | |
| --- | --- | --- | --- | --- | --- |
|  |  | Index test outcome | |  |  |
|  |  | positive | negative |  |  |
| Reference test outcome | positive | 7 | 0 | 1.00 | sensitivity |
|  | negative | 41 | 134 | 0.77 | specificity |
|  |  | 0.15 | 1.00 |  |  |
|  |  | positive predictive value | negative predictive value |  |  |

Supplemental table 2: Cross tabulation of conventional teledermatology with additional teledermoscopy test performance

| Index test: conventional teledermatology plus dermoscopic images; Reference test: face to face encounter and histopathology; total valid n=192 | | | | | |
| --- | --- | --- | --- | --- | --- |
|  |  | Index test outcome | |  |  |
|  |  | positive | negative |  |  |
| Reference test outcome | positive | 7 | 0 | 1.00 | sensitivity |
|  | negative | 28 | 157 | 0.85 | specificity |
|  |  | 0.20 | 1.00 |  |  |
|  |  | positive predictive value | negative predictive value |  |  |
